# Effects of human–machine interaction on employee’s learning: A contingent perspective

**DOI:** 10.3389/fpsyg.2022.876933

**Published:** 2022-09-07

**Authors:** Wang Sen, Zhao Hong, Zhu Xiaomei

**Affiliations:** ^1^Department of Business Administration, School of Management, Beijing Union University, Beijing, China; ^2^Department of Business Administration, School of Business Administration, Nanjing University of Finance and Economics, Nanjing, China

**Keywords:** human–machine interaction, employee vitality, employee learning, job characteristics, competence perception

## Abstract

The popularization of intelligent machines such as service robot and industrial robot will make human–machine interaction, an essential work mode. This requires employees to adapt to the new work content through learning. However, the research involved human–machine interaction that how influences the employee’s learning is still rarely. This paper was to reveal the relationship between human–machine interaction and employee’s learning from the perspective of job characteristics and competence perception of employees. We sent questionnaire to 500 employees from 100 artificial intelligence companies in China and received 319 valid and complete responses. Then, we adopted a hierarchical regression for the test. Empirical results show that human–machine interaction has a U-shaped curvilinear relationship with employee learning, and employee’s vitality mediates the curvilinear relationship. In addition, job characteristics (skill variety and job autonomy) moderate the U-shaped curvilinear relationship between human–machine interaction and employee’s vitality, especially the results of moderating effects varying with employee’s competence perception. Exploring the mechanism of the effect of human–machine interaction on employee’s learning enriches the socially embedded model. Moreover, it provides managerial implications how to enhance individual adaptability with the introduction of AI into firms. However, our research focuses more on the impact of human–machine interaction on employees at the initial stage of AI development, and the level of machine intelligence in various industries will reach a high degree of autonomy in the future. The future research can explore the impact of human–machine interaction on individual’s behavior at different stages, and the results may vary depending on the technologies mastered by different individuals. The study has theoretical and practical significance to human–machine interaction literature by underscoring the important of individual’s behavior among individuals with different skills.

## Introduction

With the advent of digital era, the popularization of artificial intelligent (AI) such as service robot, freight robot, and industrial robot has presented a new type of office landscape (Cameron, 2019; Hentout et al., 2019). Human–machine interaction is becoming more and more frequent and necessary. Some scholars contend that collaborative robots can assist workers and improve their safety and productivity (Hancock et al., 2011; Visser and Parasuraman, 2011; Rahwan et al., 2019). Consequently, employees are willing to learn to use new smart devices and technologies (Mahzoon et al., 2019). For example, through investigations and interviews from the firms applied AI, we find that the staff cooperating effectively with food delivery robots could improve work efficiency by more than 70%. However, other scholars have pointed out that many employees are afraid of robots to take away their jobs if they cannot learn to update their skills effectively (Zlotowski et al., 2017). Unfortunately, we have insufficient knowledge on how human–machine interaction influences employee’s learning. Studying the relationship between human–machine interaction and employee’s learning not only ensures the employees use the AI technologies effectively and confidently, but also keeps them thrive in a quickly changing workplace (Rampersad, 2020).

This work contributes to employee’s learning, mainly from thriving at work of Spreitzer et al. (2005). Based on the socially embedded model of thriving at work, learning is shaped by the context in which individuals are embedded (Sonnentag and Fritz, 2007; Spreitzer et al., 2010), including contextual features and available resources (Niessen et al., 2010). Contextual features, such as job stressors (Paulsson et al., 2005) and job demands (Bakker and Demerouti, 2007; Alikaj et al., 2021), have an impact on employee’s learning (LePine et al., 2005). Additionally, available resources have been supported for positive meaning at work, and the experience of positive meaning increases the employees’ feelings of learning (Prem et al., 2016).

With the introduction of artificial intelligence into firms, human–machine interaction changes technological and working context. Thus, employees need to be attuned to new job demands, establish, and adapt to emotional and relational resources with intelligent machines to influence their learning furtherly. There are two different points about the influences. On the one hand, employees worry about being replaced by machines, thus pay more psychological costs, and cause the anxiety and panic from job transfer (Salanova and Schaufeli, 2008), which are not conducive to establishing positive emotional relationship and in turn inhibit employee’s learning (Waschull et al., 2020). On the other hand, intelligent machines could replace employees to complete simple and repetitive work, thus improve work efficiency, in turn strengthen the positive emotional relationship between human and machines, and promote employee’s learning (Deming, 2017). These two opposite emotions that simultaneously act on employee’s learning may produce complex results, and this provides a theoretical basis and non-linear perspective to explore the relationship between human–machine interaction and employee’s learning.

Furthermore, whether the employee’s learning can be enhanced also depends on the working situation (Bakker and Demerouti, 2007). As a component of external situation, job characteristics may impact employee’s learning (Ryan and Deci, 2000). Hackman and Oldham (1976) referred job characteristics model, pointing out that skill variety, job autonomy, and other job characteristics affected job satisfaction. Oldham and Cummings (1996) confirmed that employees with diverse work skills could facilitate the generation of new ideas. Prem et al. (2016) found that employees who have the autonomy to decide how to do their work and depose their time could be benefited to them learn new knowledge. The application of artificial intelligence in enterprises has brought unprecedented challenges to employees. However, employees with skill variety and job autonomy are tended to explore new technologies positively and more apt to establish a harmonious emotional relationship with intelligent machines. Therefore, skill variety and job autonomy may influence the relationship between human–machine interaction and employee’s learning.

In addition, employees in the same position may response differently to the external environment changes due to their individual difference. Based on self-determination theory, competence is defined as feeling effective in one’s environment and influences individual psychological and behavioral choices (Deci and Ryan, 1985, 2000). Employees with high competence perception can interact with the environment smoothly and prefer to choose challenging targets to test and expand their skills (Howard et al., 2017). With the introduction of AI into firms, high-skill (Taks et al., 2014), unconventional, and knowledge-intensive works (Haenlein and Kaplan, 2019) are in great demand (Giselle, 2020). Employees with high competence perception could quickly adapt to the changes in work requirements involved human–machine interaction. Therefore, employees with high level of competence perception could be not sensitive to the relationship between human–machine interaction and employee’s learning moderated by job characteristics (i.e., skill variety and job autonomy). However, employee with low level of competence perception may make the moderating effect of job characteristics on the relationship between human–machine interaction and employee’s learning more prominent. Thus, the moderation effect of job characteristics varies according to different individual competence perceptions.

The main purpose of this study was to enrich the understanding of how human–machine interaction affects employee’s learning. Specifically, we study the mechanism of the relationship between human–machine interaction and employee’s learning, and how employee’s vitality meditates the relationship, and furtherly explore what are the results of job characteristics moderating the relationship of human–machine interaction with employee’s vitality. Finally, we introduce competence perception from employees to investigate the triple moderating effect of human–machine interaction, job characteristics, and competence perception on vitality.

This study makes three important contributions. First, we extend the application of socially embedded model in AI scenarios and enrich the literature of thriving at work. The socially embedded model describes how work situations and relational resources stimulate employees’ learning (Spreitzer et al., 2005). However, with the development and popularization of AI, cooperation between human and machine changes the work situations and relational resources (Blanchard et al., 2018) and could influence employee’s learning differently. Thus, we introduce the concept of human–machine interaction and point out that human–machine interaction affects employees’ emotional resources and has a U-shaped curvilinear relationship with employee’s learning.

Second, we reveal the internal mechanism of the relationship between human–machine interaction and employee’s learning. Factors that affect employee’s learning come from the work situations, job characteristics, and their abilities (Porath et al., 2012). This study argues that vitality, that is a sense of energy or passion for work, also influences employee’s learning. Meanwhile, the introduction of AI technology into firms inspires employees’ passion for work. Thus, human–machine interaction affects employee’s learning by changing their vitality. Our study enriches the psychological mechanism of influencing employee learning.

Third, we explore the boundary conditions for the effect of human–machine interaction on employee’s vitality. From the perspective of job characteristics, we find that both skill variety and job autonomy enhance the relationship between human–machine interaction and employee’s vitality. The study supports job characteristics model from Hackman and Oldham (1976), and that job characteristics have an impact on employee’s psychology. Furtherly, considering that personal psychology is not only affected by job characteristics, but also has a close relationship with one’s own conditions, we introduce competence perception and point out that competence perception moderates the moderating effect of job characteristics. This paper explores the triple-interactive effects of human–machine interaction, job characteristics, and competence perception on individual’s behavior, expanding the research on the relationship between human–machine interaction and employee’s psychological behavior.

The rest of this article is organized as follows. First, we review theoretical background and propose hypothesis development. Second, we develop the research method to collect the data and measure the variables analyzed in this study. Third, we provide the results of hypotheses testing. Fourth, we discuss the implications of our research and identify the limitations and promising areas for future research. Finally, we conclude the conclusion.

## Theoretical background and hypothesis development

Employee’s learning is not only rooted in individual independent thoughts, but also in social systems and formulated in the process of social interactions (Wenger, 1998). Gherardi et al. (1998) state that learning often occurs in the social interaction among employees, and the emotional and relational resources affect the frequency and initiative of interpersonal interaction (Brown and Duguid, 1991). As a new work style, human–machine interaction requires constant social interaction between human and AI. Different human emotions and feelings are generated during interaction with machines (Mahzoon et al., 2019), thereby affecting employee’s learning. Based on the bivariate model of positive and negative emotions, the study explains the effect of human–machine interaction on employee’s learning, portraying the process of employees’ emotional changes.

The equilibrium spatial model (ESM), a typical representative of the bivariate model of emotions, demonstrates that positive and negative emotions are two independent variables, and one of the relations between the two emotions is reflected in co-activation, implying that the changes in one emotion are accompanied by parallel changes in the other emotion and can occur simultaneously (Uphill et al., 2019). Individual psychological change is the process of perceiving both positive and negative emotions. The superposition of the two emotions is the total effect of human–machine interaction on employee’s learning. According to the superposition principle of non-linear causes proposed by Haans et al. (2016), the superposition of these opposite effects of two emotions may lead to a U-shaped relationship between human–machine interaction and employee’s learning. Based on the moderating effect analysis (Haans et al., 2016), we divide the intensity of human–machine interaction into two scenarios, low (low to medium) and high (medium to high) in the theoretical deduction.

In the scenario of low to medium, the degree of human–machine interaction is low at the initial stage of AI introduction into employees’ work because of employees lacking the experience with new technologies and devices. The work involved human–machine interaction leads to a sudden increase in the requirement for work skills, and the employees need to transform service-based manual labor to creative intellectual labor (Manyika et al., 2017). Employees are needed to constant exploration and friction with machines. Acemoglu and Restrepo (2018) document that the perception of AI is reflected in terms of the uncertainty and uncontrolled ability of future work, such as whether machines will undertake more replacement work or even replace individual jobs, thus putting themselves at the risk of unemployment. This panic overtakes the positive emotions that smart machines can trigger high productivity and dominates in the human brain. When negative emotions dominate, it is difficult for employees to focus on performing their job duties. They are tired of exploring new technical knowledge, and not conducive to developing intimate partnerships with machines, thereby decreasing employee’s learning.

In the scenario of moderate to high, there is a tipping point to human–machine interaction after which human–machine interaction can enhance employee’s learning greatly. In this situation, through unceasing running-in with machines, individuals mainly undertake creative work, but repetitive and systematic work is taken by machines (Deming, 2017). Thus, employees’ positive emotions outweigh the negative emotions, thereby improving individual productivity significantly. Individuals gradually recognize that machines acting as aids could help them more focus on some creative work, motivating employees to keep exploring given areas and enhancing learning ability (Spreitzer and Porath, 2013). As the level of human–machine interaction gradually increases, employees also could form close collaborative relationships with machines and view AI as partners and assistants (Markoff, 2016), in turn stimulating employee’s learning. Therefore, with the enhancement of man–machine interaction, the influence of man–machine interaction on employee’s learning shows a trend of decrease first and then increase. Accordingly, we propose Hypothesis 1.

H1: Human–machine interaction is curvilinearly (taking a U-shape) related to employee’s learning.

Miller and Stiver (1997) suggest that vitality is generated through constant interaction with others and emphasize that the associated relationships among individuals could stimulate individual vitality. Human–machine interaction is a new type of cooperation relationship between human and machines (Michael and Sabrina, 2021), having the impact on individual vitality. In the early stage of AI application, the dominance of individual negative emotions reduces the vitality of employees. As the degree of human–machine interaction deepens, machines can gradually replace repetitive work contents, so that individuals can extricate themselves from tedious work and focus on creative work (van Esch et al., 2021). Therefore, individuals’ positive emotions take over and they will feel energized (Ryan and Deci, 2000). Meanwhile, when people only focus on their tasks, they are more likely to perform effectively (Brown and Ryan, 2003). Thus, when individuals successfully complete their work, they may feel a sense of accomplishment, in turn increasing individual vitality.

Vitality is a psychological state, showing a feeling of having positive energy (Nix et al., 1999) and facilitating individual learning to some extent, because vitalized individuals have higher instantaneous attentions and thinking capacities to facilitate individual learning (Fredrickson, 2003). What is more, negative emotions could narrow the range of thinking behaviors, whereas positive emotions could promote aggressive behaviors and increase readiness for action (Forgas, 2010). In sum, individual vitality can motivate individuals to keep improving and learning. Therefore, human–machine interaction can affect individual learning by influencing employees’ individual vitality. Thus, we propose Hypothesis 2.

H2: Individual vitality mediates the relationship between human–machine interaction and employee’s learning.

All people have the capacity to pursue learning and development, but whether they can effectively achieve this pursuit also depends on their job characteristics (Ryan and Deci, 2000). According to Hedström and Swedberg (1998) point, job characteristics and the resources generated in working could influence individual learning through situational mechanisms. We choose two types of job characteristics (i.e., skill variety and job autonomy) to research the moderating effects of job characteristics on the relationship between human–machine interaction and individual vitality. In particular, according to Kahya (2007), skill variety refers to the extent to which the job requires the employee to draw from a number of different skills and abilities as well as upon a range of knowledge (Noefer et al., 2009), which will reduce boredom and increase job satisfaction and motivation (Kemboi et al., 2013), thereby influencing employee’s learning. Scholars have argued that job autonomy, the ability to decide when, where, and how the job is to be done, most likely has an effect on employees’ well-being (Thompson and Prottas, 2006). Employees with high job autonomy experience less stress and high career satisfaction (Parasuraman and Alutto, 1984) and finally facilitate their learning.

Under the condition of human–machine interaction level from low to medium, the negative emotions brought by man–machine collaboration are dominant, leading to the gradual decrease in individual vitality. If the work has diverse characteristics, it is difficult for employees to focus on work. Moreover, the working mode of human–machine interaction requires individuals to reduce the stickiness to the previous variety of work, in turn enhancing the inadaptability of employees and thus decreasing individual vitality. Simultaneously, as the introduction of intelligent machines may lead to individuals unable to arrange their work like before, therefore the more autonomy employees possess in their work, the more confused they will be (Batt and Valcour, 2003), furtherly leading to increase negative emotions and decrease individual vitality. Hence, compared with low job autonomy, human–machine interaction is more likely to inhibit individual vitality under high job autonomy.

Under the condition of human–machine interaction level from moderate to high interval, the positive emotions generated by human–machine interaction are enhanced and amplified. As analyzed in the above, with the continuous strengthening of human–machine interaction, employees’ emotions could reverse from the negative emotions of panic at the beginning of human–machine interaction to the positive emotions generated by human–machine interaction replacing their own complicated tasks. Furthermore, the dominated positive emotions trigger individual vitality. Meanwhile, employee with diverse skills also will further stimulate their vitality (Morris and Venkatesh, 2010). According to the job characteristics model, skill variety is not only an expansion of the horizontal quantity of work, but also an expansion of the vertical depth (Noefer et al., 2009). The higher degree of autonomy at work implies greater responsibility and self-determination (Thomas and Ganster, 1995). With the deepening of human–machine interaction, individual work responsibility can further enhance individual work efficiency, activate positive emotions, and relieve unemployment anxiety, thereby stimulating individual vitality. The self-determination enables employees to acquire and use new knowledge and skills to solve problems (Voydanoff, 2004), thereby enhancing individual vitality. In sum, we propose Hypotheses 3a and 3b.

H3a: Skill variety enhances the relationship between human–machine interaction and individual vitality.

H3b: Job autonomy enhances the relationship between human–machine interaction and individual vitality.

Employees with different traits under the same job characteristic conditions may react completely different to the changes in the external environment. Given that individual vitality partly depends on how competent employees feel about their jobs (Prem et al., 2017), it is necessary to introduce competence perception into research. Competence perception reflects the degree of individuals controlling over their work (Deci et al., 2017). Individuals with high competence perception are able to interact with their surroundings autonomously and prefer challenging jobs (Howard et al., 2017), whereas individuals with low competence perception have limited control over their jobs and prefer more conservative jobs (Dweck, 1999).

The non-linear moderating effect of high-skill variety is moderated by competence perception. In the low to moderate level, human–machine interaction is the early stage of the introduction of artificial intelligence. During this process, employees with high-skill variety do not know how to carry out man–machine interaction, thus reducing individual vitality. If individuals possess high competence perception, once losing control of the work of human–machine interaction, they will get frustrated and lead to reduce their vitality. By contrast, compared with employees with high competence perception, individuals with low competence perception accept new jobs passively and are not sensitive to the possible job challenges. As a result, the inhibition effect of skill variety on human–machine interaction and individual vitality is weakened. In the moderate to high level, with deepening of the human–machine interaction, employees with high-skill variety become increasingly focus and explore creative work in certain areas, thereby increasing individual vitality. However, individuals with low competence perception are more prefer to conservative work, in turn preventing them from exploring creative work in depth (Vergauwe et al., 2015). In addition, this inhibits the positive effect of high-skill variety on the relationship between human–machine interaction and individual vitality. Therefore, when individuals have low competence perception and high-skill variety, the effect of human–machine interaction on vitality is weakened.

Similarly, the moderating effect of high job autonomy is also moderated by competence perception. In the low to moderate level, human–machine interaction decreases employees’ individual vitality. High job autonomy hinders employees to schedule their own work (Hackman and Oldham, 1976). Employees with low competence perception are less impact to schedule their own work than those with high competence perception, thus weakening the negative effect of job autonomy on the relation between human–machine interaction and individual vitality. In the moderate to high level, for individuals with high job autonomy, with the deepening degree of human–machine interaction, individual work productivity can be improved and the anxiety of unemployment can be alleviated to some extent. However, for individuals with low job competence perception, it is difficult for them to interact with the machine autonomously, and thus, they are reluctant to choose challenging jobs to expand their skills. As a result, unemployment anxiety is not effectively alleviated, which further inhibits the promoting effect of high job autonomy on human–machine interaction and individual vitality. In sum, Hypotheses 4a and 4b are proposed.

H4a: The interactions of competence perception, skill variety, and human–machine interaction affect individual vitality.

H4b: The interactions of competence perception, job autonomy, and human–machine interaction affect individual vitality.

**FIGURE 1:**
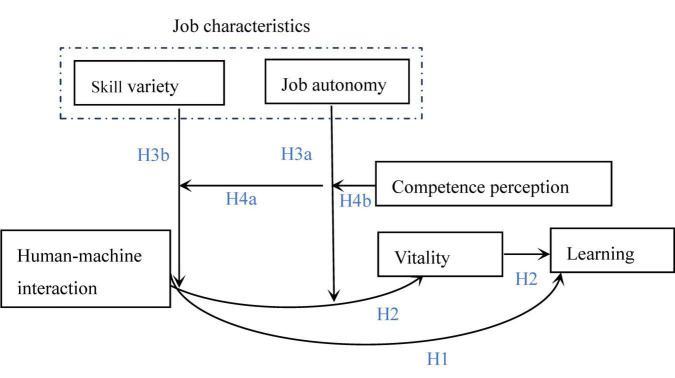
Conceptual model.

Finally, we propose a conceptual model, as illustrated in Figure 1.

## Materials and methods

### Sampling and data collection

To test our hypotheses, we employ multiple-informant survey technique and use 319 valid survey data from employees and their leaders in the firms applying AI. These firms are located in Beijing Province in China. We selected the firms in Beijing Province in China for several reasons. First, Beijing takes the leading position in AI technology and also is the technological innovation center of China, ranking first in terms of the number of new-generation AI open innovation platforms, AI-related talents, and AI patent applications among China. Second, Beijing gathers lots of mature AI application firms and vigorously promotes the construction of application scenarios. Since 2019, two batches of 40 application scenario construction projects have been released, focusing on the key areas such as digital finance, digital marketing, smart manufacturing, smart education, smart healthcare, and so on. Policy incentives spawned a large number of AI application firms. Third, with the epidemic of COVID-19, integration of AI and traditional economy is accelerating, such as unmanned delivery, online consumption, and smart economy, which greatly help China to fight against the epidemic. With the highlights of the competitive advantages of AI technology, many firms in Beijing have begun to apply AI technology, including unmanned distribution, remote office, etc. Therefore, firms applying AI in Beijing Province provide ideal contexts to investigate how human–machine interaction affects employees’ learning.

There were two independent researchers who first developed the questionnaire combined with research topics, and we discussed any divergence between each other until reached a consensus. We did a preresearch before the formal questionnaire research. Since the preresearch objects need not be representative, but be relevant (Babbie, 2010), we conducted in-depth interviews with 3 firms in different industries that we tracked for a long time, and these firms all have introduced AI-related equipment or technology, including a bank, a high-tech firm, and a communication firm. Additionally, the interview mainly focused on the changes in work content, psychology, and behavioral perception of employees after the introduction of AI into firms. After the interview, we issued questionnaires to 20 participants as a pilot test. We asked these respondents not only to answer all the questionnaire items but also to provide feedback about design and wording. According to the suggestions from the feedback, we made detailed revision to the questionnaire and finally formed a formal questionnaire.

Then, we cooperated with a management consulting firm to help us randomly select 500 AI application firms and issued questionnaires with the assistance of administrative agencies. In addition, government administrative agencies were responsible to collect the questionnaires. We recruited experienced interviewers and trained them before conducting the on-site survey. To encourage the participants to keep neutrality and objectivity, we explained them that this survey is for academic use only and we reassured the confidentiality of the data collected. What is more, to protect the participants from any adverse consequence, we carefully crafted the questionnaire with no sensitive information involved, such as firm name, code, location, and contact number. Participants in the survey also need to meet the following criteria: (1) the firms have introduced AI-related equipment or technology. (2) Participants’ leaders are willing to cooperate with the survey. (3) Participants keep the contents of the survey confidential and do not inform colleagues.

Furtherly, to ensure the quality of the collected data, we distributed two versions of questionnaires in different times. The A version of questionnaire was filled out by employees, including human–machine interaction, vitality, and other variables involved questions. A total of 500 questionnaires were distributed (459 were returned). Then, 2 months later, we handed out the B version of questionnaire to the previous employees and asked them to answer their competence perception and learning involved items. We also invited their leaders to answer subordinates’ skill variety and job autonomy-related items. After deleting invalid and unmatched questionnaires, we received 319 useable questionnaires, for an effective response rate of 63.8%.

### Measures

We use five-point Likert (1 = “strongly disagree” and 5 = “strongly agree”) scales to operationalize our constructs.

#### Dependent variable

##### Employee’s learning

It is measured by four items adapted from Porath et al. (2012), including “I am becoming more mature, I am improving, I am learning, and I am growing.” Participants are instructed to evaluate each statement against their own situation on a five-point Likert scale (1 = strongly disagree and 5 = strongly agree), and higher scores representing a greater degree of learning.

#### Independent variables

##### Employee’s vitality

Employees are asked to report whether they feel enthusiastic about their work. It is measured by four items adapted from Porath et al. (2012), including “I often feel full of vitality, I often feel energetic, I often feel clear in my thinking, and I often feel sharp in my thinking.”

##### Skill variety

It is measured by three items adapted from Sims et al. (1976). Additionally, these items are answered by participants’ leaders, namely, “The degree of diversity of subordinate’s work in the context of AI, Repetition of subordinate’s work in the context of AI, and subordinate’s chances of doing many different things in the context of AI.”

##### Job autonomy

We measure it with scales adapted from Karasek (1979), combined with the job characteristics of employees in the AI context, including the following three items: “the extent to which the subordinate can work freely and autonomously in the context of artificial intelligence, the extent to which the subordinate can adjust my work schedule in the context of artificial intelligence, and the extent to which the subordinate can adjust my work schedule in the context of artificial intelligence.”

Competence perception: It is used to assess psychological feelings of employees about their competency in performing specific tasks. The items are chosen from the scales developed by Sheldon et al. (2001), including “I am competent at my job, I often experience a sense of accomplishment at work,” and so on.

Human–machine interaction: The variable followed a three-step procedure for scale development. In the first step, we use literature deductive method and in-depth interview method to determine the initial questionnaire items. In this study, we first review the existing research on human–machine interaction defined as a collaborative relation between employees and intelligent machines. Considering that the work of intelligent machines is highly correlated with computer applications, we refer to the scale of computer application developed by Medcof (1996). Thus, we use the artificial intelligence equipment application to replace the computer application in the original scale to represent the degree of interaction between human and machines. The measurement to the relationship between employees and intelligent machines refers to the friendship subscale from the job characteristics scale developed by Sims et al. (1976). Based on the literature review, we conduct in-depth interviews with 20 employees from three AI firms and then revise the scale items to ensure them applicable to the human–machine interaction context. In the second step, to verify the validity of the questionnaire, we implement an exploratory study and furtherly revise the measurement items of scale. We verify the 100 valid questionnaires collected first and conduct an exploratory factor analysis using Statistical Packages for the Social Sciences (SPSS 22.0). These results indicate that the reliability and validity of the scale items reach the standards when the factor loading is greater than 0.5, the KMO test is greater than 0.7, and the Cronbach’s α is greater than 0.6. In the third step, validation factor analysis is conducted to validate the scale. Therefore, the final scale items of human–machine interaction included “(1) I use AI devices at work for a long time; (2) I like AI devices at work; (3) I consider AI devices as my work partners; (4) I often introduce the AI devices used at work to my family or friends.” Participants are instructed to evaluate each statement against their own situation on a five-point Likert scale (1 = strongly disagree and 5 = strongly agree), with higher scores representing a greater degree of human–machine cooperation relationship.

In addition, we control for the effects of demographic characteristic variables such as gender, age, position, job title, education, and income.

## Analysis and results

### Tests of reliability and validity

The coefficient Cronbach’s α of the six core variables all ranged from 0.615 to 0.886, indicating highly internal consistency. The CR values are greater than 0.7, showing each construct possessing a good combined reliability. The AVE value for each construct is greater than 0.5, in support of good convergent validity (Table 1).

**TABLE 1:** Variable reliability and convergent validity.

Variable	Cronbach’s α	CR	AVE
Human–machine interaction	0.730	0.839	0.568
Vitality	0.857	0.838	0.564
Learning	0.886	0.797	0.598
skill variety	0.615	0.795	0.568
Job autonomy	0.746	0.851	0.656
Competence perception	0.629	0.795	0.566

### Tests for common method bias

In addition, common method bias (CMB) will arise from using data from the same side (Podsakoff et al., 2003). To address the potential problem, we first managed the survey process using a pretest and reverse-coded items were included the questionnaires (Malhotra et al., 2006). Furtherly, all of our questionnaires were filled out by the employees and their leaders. The measurement of independent variables and dependent variables was obtained from different sources. Second, we adopted a Harman’s single factor test by entering all the principal constructs into a principal component factor analysis (Table 2). Through the analysis, the largest variance explained by the first factor is 12.027%, which is less than 30% (Harman, 1976). Overall, these findings suggest that CMB is not a concern in our study.

**TABLE 2:** Total variance explained with cumulative percentage of components.

Total variance explained									

Ingredients	Initial eigenvalue			Extraction of the sum of squares of loads			Sum of squared rotating loads		
			
	Aggregate	Percentage variance	Cumulative percentage	Aggregate	Percentage variance	Cumulative percentage	Aggregate	Percentage variance	Cumulative percentage
1	4.983	23.727	23.727	4.983	23.727	23.727	2.526	12.027	12.027
2	2.270	10.810	34.537	2.270	10.810	34.537	2.307	10.987	23.014
3	1.814	8.640	43.176	1.814	8.640	43.176	2.232	10.626	33.640
4	1.395	6.644	49.821	1.395	6.644	49.821	2.177	10.366	44.006
5	1.176	5.600	55.421	1.176	5.600	55.421	1.760	8.381	52.387
6	1.097	5.222	60.643	1.097	5.222	60.643	1.734	8.256	60.643

The extraction method is a principal component analysis.

### Descriptive statistics and correlations

The descriptive statistics (mean and standard deviation) of the variables and the correlation coefficients between the variables are presented in Table 3. The correlations of employee’s learning with the main research variables, including vitality, skill variety, and job autonomy, are significant. However, the linear relation between the learning and human–machine interaction is not significant, so it is tentatively judged to be non-linear.

**TABLE 3:** Descriptive statistics and correlation analysis.

Variable	1	2	3	4	5	6
(1) Gender	1					
(2) Age	–0.216**	1				
(3) Position	324**	0.037	1			
(4) Job title	0.009	–0.145**	0.354**	1		
(5) Education	0.056	0.052	0.155**	0.203**	1	
(6) Income	–0.167**	0.034	0.093	–0.230**	–0.380**	1
(7) Human–machine interaction	–0.039	–0.163**	0.152**	–0.066	–0.032	0.107
(8) Vitality	–0.017	–0.150**	0.084	0.009	–0.083	0.180**
(9) Learning	0.044	–0.173**	0.084	–0.086	–0.164**	0.183**
(10) Skill variety	–0.144**	–0.146**	0.171**	0.082	–0.122*	0.298**
(11) Job autonomy	–0.014	0.057	0.162**	–0.058	–0.122*	0.290**
(12) Competence perception	–0.004	–0.013	0.06	0.015	–0.154**	0.211**
Average	1.451	3.777	3.292	3.674	2.981	4.445
SD	0.498	1.078	1.770	0.668	0.649	0.833

**Variable**	**7**	**8**	**9**	**10**	**11**	**12**

(7) Human–machine interaction	1					
(8) Vitality	0.288	1				
(9) Learning	0.330	0.359**	1			
(10) Skill variety	0.200**	0.253**	0.246**	1		
(11) Job autonomy	0.189**	0.046	0.121	0.256**	1	
(12) Competence perception	0.401**	0.442**	0.288**	0.222**	0.168**	1
Average	3.918	4.158	3.815	3.171	3.544	4.111
SD	0.597	0.482	0.376	0.583	0.650	0.483

+p < 0.1, *p < 0.05, **p < 0.01. Two-tailed test.

### Hypothesis testing

#### Analysis of main regression

To test the effect of human–machine interaction on employee’s learning, we adopted a hierarchical regression method and the procedure for non-linear conditioning test suggested by Aiken and West (1991). We entered the control variables and independent variables hierarchically (Table 4). Model 1 provides a regression analysis of the relation between control variables and individual learning. Based on this analysis, Model 2 adds human–machine interaction and its quadratic term to test the non-linear effect of human–machine interaction on learning. The results reveal that coefficient of human–machine interaction is significantly negative (β = –0.556, *p* < 0.01), and quadratic term of human–machine interaction is significantly positive (β = 0.104, *p* < 0.001). Thus, these results indicate a U-shaped non-linear relationship between human–machine interaction and employee’s learning. Hypothesis 1 receives support.

**TABLE 4:** Results of regression analysis.

Variable	Learning		
	Model 1	Model 2	Model 3
Constant	4.053***	4.507***	3.1***
Sex	0.067	0.062	0.065
Age	–0.061**	–0.032^+^	–0.045*
Position	0.036**	0.02	0.031*
Job title	–0.068*	–0.046	–0.069*
Education	–0.068 ^+^	–0.065*	–0.064^+^
Income	0.052 ^+^	0.041	0.028
Human–machine interaction		–0.556**	
Square of human–machine interaction		0.104***	
Vitality			0.241***
*R* ^2^	0.1	0.203	0.19
Adjusted R^2^	0.082	0.183	0.171
*F*	5.755**	9.878***	10.392***

+p < 0.1, *p < 0.05, **p < 0.01, ***p < 0.001. Two-tailed test.

#### Analysis of the mediating effects of vitality

Models 3 and 5 are used to test the mediating effect of vitality on the relation between human–machine interaction and learning (Table 5). The results revealed that the effect of vitality on employee’s learning is significantly positive (β = 0.241, *p* < 0.001). The coefficient of human–machine interaction is significantly negative (β = –0.748, *p* < 0.01), whereas its secondary-order coefficient is significantly positive (β = 0.136, *p* < 0.01), tentatively indicating the mediating effect of vitality.

**TABLE 5:** Results of the analysis of mediating and moderating effects.

Variable	Vitality					
	
	Model 4	Model 5	Model 6	Model 7	Model 8	Model 9
Constant	3.95***	4.645*	7.129***	4.226***	5.736***	5.376***
Sex	0.004	–0.001	–0.002	–0.007	0.008	0.013
Age	–0.07**	–0.035	–0.028	–0.043^+^	–0.024	–0.035
Position	0.021	0.002	0.002	0.011	0.005	0.008
Job title	0.002	0.028	0.035	0.012	0.035	0.011
Education	–0.017	–0.013	–0.029	0.007	–0.019	0.009
Income	0.099**	0.085*	0.079**	0.054^+^	0.099**	0.075**
Human–machine interaction		–0.748**	–2.034***	–1.122*	–1.241***	–1.539***
Square of human–machine interaction		0.136**	0.292***	0.158**	0.196***	0.214***
skill variety			0.041	0.097		
Human–machine interaction × skill variety			–2.577***	0.918		
Square of human–machine interaction × skill variety			0.307**	–0.116		
Job autonomy					–0.065	–0.08
Human–machine interaction × job autonomy					–1.203***	0.316
Square of human–machine interaction × job autonomy					0.138**	–0.044
Competence perception				0.325***		0.351***
skill variety × competence perception				0.103		
Human–machine interaction × skill variety × competence perception				1.596**		
Square of human–machine interaction × skill variety × competence perception				–0.176**		
Job autonomy × competence perception						–0.002
Human–machine interaction × job autonomy × competence perception						1.147**
Square of human–machine interaction × job autonomy × competence perception						–0.134**
*R* ^2^	0.063	0.155	0.199	0.317	0.21	0.340
Adjusted *R*^2^	0.044	0.133	0.17	0.283	0.182	0.308
*F*	3.467**	7.094***	6.917***	9.383***	7.422***	10.424***

+p < 0.1, *p < 0.05, **p < 0.01, ***p < 0.001. Two-tailed test.

Furthermore, we examined the mediating effect of individual vitality using the bias-corrected percentile bootstrap method (Preacher and Hayes, 2008). The results (Table 6) indicate the indirect effect of human–machine interaction through the mediation of vitality is 0.008, with a confidence interval (CI) of [–0.003, –0.013], which does not contain 0 and therefore is significant, indicating that the mediating effect of vitality on the relationship between human–machine interaction and learning is positive and significant. We get support for Hypothesis 2.

**TABLE 6:** Tests for mediating effects of vitality.

Square of human–machine interaction → total effect of learning (95% confidence interval, CI)	Square of human–machine interaction → direct effect of learning (95% CI)	Square of human–machine interaction → indirect effects of learning (95% CI)
0.032*** [0.023,0.041]	0.024*** [0.015,0.033]	0.008*** [0.003,0.013]

***p < 0.001. Two-tailed test. Bootstrap = 5000.

#### Analysis of the moderating effect of job characteristics

Models 6 tests the moderating effect of skill variety on the relation between human–machine interaction and employee’s vitality. The moderating effect of skill variety on human–machine interaction and employee’s vitality relationship is negative and significant (β = –2.577, *p* < 0.001), as well as moderating effect of skill variety on quadratic term of human–machine interaction and employee’s vitality is positively significant (β = 0.307, *p* < 0.01). Models 8 tests the moderating effect of job autonomy, showing the result of moderating effect of job autonomy on the relationship between human–machine interaction and employee’s vitality is negative and significant (β = –1.203, *p* < 0.001), but the result of moderating effect of job autonomy on the relationship between quadratic term of human–machine interaction and employee’s vitality is positively significant (β = 0.138, *p* < 0.01). Therefore, the results support Hypotheses 3a and 4a.

Subsequently, we performed simple estimates of slope of the curve and limited the mean plus or minus one standard deviation of the moderating variable skill variety and job autonomy, respectively (Table 7). The results of the skill variety moderating effect reveal a U-shaped curvilinear relationship between human–machine interaction and individual vitality for both high-skill variety (β = 0.03, *p* < 0.001) and low-skill variety (β = 0.041, *p* < 0.001). The results of moderating effect of job autonomy reveal a quadratic curve state for the relation between human–machine interaction and vitality under both high job autonomy (β = 0.027, *p* < 0.01) and low job autonomy (β = 0.044, *p* < 0.001). Thereby, both Hypothesis 3a and 3b are supported.

**TABLE 7:** Simple estimates of the slope of the moderating effect.

Moderating variables		Simple estimate of slope	SE	*T*-value	*P*-value	95% confidence interval (CI)	
					
						Lower bound	Upper bound
skill variety	+SD	0.030***	0.008	3.594	0.000	0.013	0.046
	–SD	0.041***	0.009	4.737	0.000	0.024	0.058
Job autonomy	+SD	0.027**	0.008	3.267	0.001	0.011	0.044
	+SD	0.044***	0.008	5.661	0.000	0.028	0.059

**p < 0.01, ***p < 0.001. Two-tailed test.

Furthermore, we separately examined the mediated effects of vitality moderated by skill variety and job autonomy. In particular, the bootstrap method test for the mediating effect of vitality is moderated by skill variety. The result reveals that the indirect effect of skill variety through vitality moderating the non-linear relation between human–machine interaction and individual learning is significant (with a 95% CI [0.003, 0.012]), as shown in Table 8. The result of the bootstrap method test for the mediating effect of vitality moderated by job autonomy reveals in Table 9 and shows that the indirect effect of job autonomy through individual vitality moderating the non-linear relation between human–machine interaction and individual learning is significant.

**TABLE 8:** Tests for mediating effects of skill variety regulation.

Square of human–machine interaction × skill variety → vitality (95% CI)	Square of human–machine interaction → direct effect of learning (95% CI)	Square of human–machine interaction → indirect effects of learning (95% CI)
–0.014 [–0.041,0.014]	0.024*** [0.015,0.033]	0.008*** [0.003,0.012]

***p < 0.001. Two-tailed test. Bootstrap = 5000.

**TABLE 9:** Tests for mediating effects of job autonomy regulation.

Square of human–machine interaction × job autonomy → vitality (95% CI)	Square of human–machine interaction → direct effect of learning (95% CI)	Square of human–machine interaction → indirect effects of learning (95% CI)
–0.013 [–0.028,0.013]	0.024*** [0.015,0.033]	0.008*** [0.003,0.012]

***p < 0.001. Two-tailed test. Bootstrap = 5000.

#### Analysis of the moderating effect of competence perception

We test the moderating effect of competence perception moderated job characteristics in the relationship between human–machine interaction and individual vitality. Model 7 in Table 5 indicates that the product term of human–machine interaction, skill variety, and competence perception is significant (β = 1.596, *p* < 0.01), and the product result of the quadratic term of human–machine interaction, skill variety, and competence perception is negatively significant (β = –0.176, *p* < 0.01). Model 9 in Table 5 indicates that the product of human–machine interaction, job autonomy, and competence perception is significant (β = 1.147, *p* < 0.01), and the product of quadratic term of human–machine interaction, job autonomy, and competence perception is negatively significant (β = –0.134, *p* < 0.01).

Accordingly, we classified skill variety and competence perception into four cases, namely, (1) high-skill variety and high competence perception, (2) high-skill variety and low competence perception, (3) low-skill variety and high competence perception, and (4) low-skill variety and low competence perception, for simple slope estimation. Furthermore, we also classified job autonomy and competence perception into four separate cases (Table 10). The results reveal that the joint moderating effect of low-skill variety and high competence perception does not pass the test (β = 0.022, 95% CI [–0.002, 0.046]), rejecting Hypothesis 4a. The joint moderating effect of high job autonomy and low competence perception is significant (β = –0.043, 95% CI [–0.068, –0.017]), thereby verifying Hypothesis 4b.

**TABLE 10:** Simple estimates of the slope of the moderating effect.

Moderating variables	Simple estimate of slope	SE	*T*-value	*P*-value	95% confidence interval (CI)	
					
					Lower bound	Upper bound
High-skill variety, high competence perception	0.015	0.01	1.541	0.124	–0.004	0.034
High-skill variety, low competence perception	–0.017	0.013	–1.367	0.173	–0.042	0.008
Low-skill variety, high competence perception	0.022	0.012	1.794	0.074	–0.002	0.046
Low-skill variety, low competence perception	0.024*	0.01	2.368	0.018	0.004	0.045
High job autonomy, high competence perception	0.016	0.009	1.758	0.08	–0.002	0.034
High job autonomy, low competence perception	–0.043**	0.013	–3.266	0.001	–0.068	–0.017
Low job autonomy, high competence perception	0.021	0.011	1.97	0.05	0.000	0.043
Low job autonomy, low competence perception	0.025**	0.009	2.927	0.004	0.008	0.042

*p < 0.05, **p < 0.01. Two-tailed test.

## Discussion

The main purpose of this study was to enrich the understanding of how human–machine interaction affects employee’s learning in the AI context. Based on socially embedded model, we investigate the relationship between human–machine interaction and employee’s learning. The empirical results suggest that the following: First, human–machine interaction has a U-shaped effect on employee’s learning. With the deepening of human–machine interaction, employee’s learning shows a trend of first decreasing and then increasing. In addition, we reveal the mechanism that how human–machine interaction affects learning. Second, employee’s vitality mediates the relationship between human–machine interaction and employee’s learning. Then, by identifying job characteristics as the critical contingent factor, we explore the moderating effect of skill variety and job autonomy on human–machine interaction with employee’s vitality. Third, both skill variety and job autonomy enhance the relationship between human–machine interaction and vitality. Finally, considering about the individual differences, we explore how the interaction between competence perception, job characteristics, and human–machine interaction affects employee’s vitality. The results reveal that under the scenario of low competence perception and high job autonomy, the negative effect of human–machine interaction on individual vitality diminishes at the beginning of the introduction of AI. As human–machine interaction deepens, the positive effect of human–machine interaction on individual vitality is diminishing.

### Theoretical and contributions

This study contributes to the existing employee’s learning literature mainly reflecting in two aspects. First, based on the socially embedded model, the work conditions affect employee’s learning (Spreitzer et al., 2010). With the development and popularization of AI, human–machine interaction changes the work situations (Kulic and Croft, 2007; Hwang et al., 2013; Blanchard et al., 2018), and thus, human–machine interaction indirectly affects employee’s learning. We introduce human–machine interaction and appropriately supplement to the socially embedding model. Second, some scholars show that human–machine interaction may create a sense of threat, reducing employees’ work enthusiasm (Riek et al., 2006; Ferrari et al., 2016; Zlotowski et al., 2017), whereas others refer that human–machine interaction could generate a sense of positive emotions among employees and improve their work efficiency (Madhavan and Wiegmann, 2007; Papadopoulos et al., 2016). Based on the bivariate emotional model, our study found that human–machine interaction can generate both positive and negative emotions among employees, with a U-shaped impact on employee’s learning, and the conclusion enriches the literature on employee’s learning.

Second, we reveal the internal mechanism of the relationship between human–machine interaction and employee’s learning. Factors that affect employee’s learning mainly come from the work situations, job characteristics, transformational leadership, and their abilities (Karasekr and Theorellt, 1990; Fredrickson, 2001). From the perspective of employees’ psychology, few scholars pay attention to the impact of employees’ emotions on employees’ learning (Seo et al., 2004; Furnham, 2016). This study argues that employee’s vitality, as a sense of passion for work, also influences employee’s learning. Meanwhile, in this paper, we introduce human–machine interaction and the results show that it affects employees’ sense of passion. Thus, employee’s vitality mediates the relationship between human–machine interaction and employee’s learning. Our study enriches the psychological mechanism of employees’ learning.

Third, we explore the boundary conditions for the effect of human–machine interaction on employee’s vitality. Based on job characteristics model, we explore the moderating effect of skill variety and job autonomy on the relationship between human–machine interaction and employee’s vitality, and the results support the point that job characteristics have an impact on employee’s psychology (Hackman and Oldham, 1976; Grzywacz and Marks, 2000; Bell and Kozlowski, 2008). However, employee’s vitality is not only affected by job characteristics (Spreitzer et al., 2010), but also has a relationship with one’s psychology (Yang et al., 2022), so we introduce competence perception and study the triple-interactive effects of human–machine interaction, job characteristics, and competence perception on employee’s vitality. This study expands the research on the relationship between human–machine interaction and employee’s psychological behavior.

### Implications for management

This study reveals the U-shaped curve relation between human–machine interaction and employee’s learning in the AI context. The implications of the findings are mainly reflected in the following aspects:

First, the smooth transition of employees at the initial stage of AI introduction through training is essential. In the early stage of AI introduction, uncertainty and panic emotions lead to the dominance of employees’ negative emotions and a decrease in learning motivation. Firms should notice the emotions and behaviors of employees in the early stage of AI introduction. Firms should also improve employees’ knowledge and skills by organizing training courses, relevant lectures, and other learning forms to stimulate their learning motivation and work enthusiasm, while, for those positions that will replaced by AI, firms should change the employee to a more suitable position or train the employee to achieve a dynamic match between the new job and the employee’s knowledge and skills.

Second, firms should increase the work diversity and job autonomy step-by-step after AI introduction. With the deepening of human–machine interaction, employees will gradually be released from the boring and complicated work. At this time, it is necessary to appropriately increase the work content of employees in positions with high-skill requirements, motivate employees to continue to explore in this field, and then stimulate their learning and work enthusiasm. Simultaneously, firms should suitably increase the autonomy of employees’ positions in the process of human–machine interaction, improve the value of work, in turn to stimulate employees’ positive emotions and vitality, and achieve sustainable organizational development.

Third, employees’ competence perception on human–machine interaction should be motivated greatly. The study shows that when individuals have high competence perception and low-skill variety (or low job autonomy), human–machine interaction has weakened the negative effect on vitality in the low degree of human–machine interaction. Therefore, in the early stage of AI introduction, firms should provide positive psychological and behavioral guidance to improve high competence perception of jobs related to human–machine interaction, thereby promoting individual vitality and learning.

### Future research outlook

First, to explore the impact of human–machine interaction on individual’s behavior in different stages of AI development, nowadays, the world is still in the early stage of AI development, and the level of machine intelligence in various industries has not yet reached the degree of autonomy. With the rapid development of 5G technology, AI will certainly take a qualitative leap forward. At different stages of AI development, individuals’ cognition to human–machine interaction may change individuals’ emotion and behavior to work. Therefore, exploring the effects of human–machine interaction on individual’s behavior at different stages of AI development is a challenging topic for future research.

Second, to study the influence of human–machine interaction on individual’s behavior in different groups, a recent study indicated that the effect of human–machine interaction on individual’s behavior may vary depending on technologies mastered by different individuals. Negative emotions such as anxiety and resistance may be obvious for technically experienced and qualified engineers who are worried that they may be replaced by AI. Therefore, it is one of the directions for future research to divide individuals according to different characteristics and examine the effect of human–machine interaction by different groups on individual’s behaviors.

Third, to scientifically and reasonably measure human–machine interaction furtherly, the empirical research on human–machine interaction remains scarce. The scale items about human–machine interaction are based on the existing related studies and refer to the scale of computer application and friendship at work to develop and finish. Although the content reliability, composite reliability, and converge validity of human–machine interaction all reach the standard, there is still space for improvement to better highlight human–machine interaction in the context of AI furtherly.

Fourth, to track the relationship between human–machine interaction and individual’s behavior, in terms of empirical research, due to the constraints of research conditions and time, we only obtain 319 valid samples from 100 AI application firms in this study. But a larger sample size of firms will help to improve the applicability of the conclusions in this paper. In addition, the data acquired in this study are cross-sectional data rather than longitudinally tracked panel data, which may also affect the in-depth theoretical and practical explorations to some extent. In the future, dynamic tracking of these sample data can be carried out to deeply analyze the evolution process of the influence of human–machine interaction on individual’s behavior and explore the internal influence mechanism of both.

## Conclusion

Overall, we seek to advance our understanding of how AI influences employee’s learning in the era of digital economy. In doing so, we introduce the concept of human–machine interaction and point out that human–machine interaction has a U-shaped curvilinear relationship with employee’s learning and employee’s vitality mediates the curvilinear relationship.

Moreover, we argue that job characteristics (skill variety and job autonomy) and competence perception of employees moderate the U-shaped curvilinear relationship between human–machine interaction and employee’s vitality. As such, we advance the model of thriving at work from the perspective of human–machine interaction as well as contribute to a more holistic understanding of the employee’s learning literature. Our conceptual model addresses the relationship between human–machine interaction and employee’s learning, and it opens up many new fascinating lines of inquiry for the future research.

## Data availability statement

The raw data supporting the conclusions of this article will be made available by the authors, without undue reservation.

## Ethics statement

Written informed consent was obtained from the individual(s) for the publication of any potentially identifiable images or data included in this article.

## Author contributions

WS was responsible for reviewing theoretical literature, proposing hypotheses, conclusion, and contributions. ZH was responsible for data analysis. ZX was responsible for implications of the article. All authors agreed to be accountable for the content of the work.

## References

[B1] Acemoglu D. Restrepo P. (2018). “Artificial Intelligence, Automation and Work,” in *Presented at the MIT department of economics Working Paper.* (Cambridge, MA), 18–11. 10.3386/w24196

[B2] Aiken L. S. West S. G. (1991). *Multiple regression: Testing and interpreting interactions.* Newbury Park, CA: Sage.

[B3] Alikaj A. Ning W. Wu B. (2021). Proactive personality and creative behavior: Examining the role of thriving at work and high-involvement HR practices. *J. Bus. Psychol.* 36 857–869. 10.1007/s10869-020-09704-5

[B4] Babbie E. R. (2010). *The practice of social research. twelfth ed.* Belmont, CA: Wadsworth Cengage Learning.

[B5] Bakker A. B. Demerouti E. (2007). The job demands-resources model: State of the art. *J. Manage. Psychol.* 22 309–328. 10.1108/02683940710733115

[B6] Batt R. Valcour P. M. (2003). Human resources practices as predictors of work–family outcomes and employee turnover. *Ind. Relat.* 42 189–220. 10.1111/1468-232X.00287

[B7] Bell B. Kozlowski S. W. J. (2008). Active learning: Effects of core training design elements on self-regulatory processes, learning and adaptability. *J. Appl. Psychol.* 93 296–316. 10.1037/0021-9010.93.2.296 18361633

[B8] Blanchard A. Kosmatov N. Loulergue F. (2018). “Connected humans, connected things: AI and human intelligence in 2018,” in *Proceedings of the 2018 International Conference on High Performance Computing & Simulation, July 16-20*, Orleans.

[B9] Brown J. S. Duguid P. (1991). Organizational learning and communities of practice: Towards a unified theory of working, learning and innovation. *Organ. Sci.* 2 40–57. 10.1287/orsc.2.1.40 19642375

[B10] Brown K. W. Ryan R. M. (2003). The benefifits of being present: The role of mindfulness in psychological well-being. *Pers. Soc. Psychol.* 84 822–848. 10.1037/0022-3514.84.4.822 12703651

[B11] Cameron S. (2019). An employee’s best friend? How AI can boost employee engagement and performance. *Strateg. HR Rev.* 18 17–20. 10.1108/SHR-11-2018-0092

[B12] Deci E. L. Ryan R. M. (2000). The what and why of goal pursuits: Human needs and the self-determination of behavior. *Psychol. Inq.* 11 227–268. 10.1207/S15327965PLI1104_01

[B13] Deci E. L. Ryan R. M. (1985). “Cognitive evaluation theory,” in *Intrinsic Motivation and Self-determination in Human Behavior. Perspectives in Social Psychology* (Boston, MA: Springer US), 87–112. 10.1007/978-1-4899-2271-7_4

[B14] Deci L. E. Olafsen H. A. Ryan M. R. (2017). Self-determination theory in work organizations: The state of a science. *Ann. Rev. Organ. Psychol. Organ. Behav.* 4 19–43. 10.1146/annurev-orgpsych-032516-113108

[B15] Deming D. J. (2017). The growing importance of social skills in the labor market. *Q. J. Econ.* 132 1593–1640. 10.1093/qje/qjx022

[B16] Dweck C. S. (1999). Caution-praise can be dangerous. *Am. Educ.* 23 4–9.

[B17] Ferrari F. Paladino M. P. Jetten J. (2016). Blurring human–machine distinctions: Anthropomorphic appearance in social robots as a threat to human distinctiveness. *Int. J. Soc. Robot.* 8 287–302. 10.1007/s12369-016-0338-y

[B18] Forgas J. P. (2010). The role of emotion in social judgments: An introductory review and an affect infusion model. *Eur. J. Soc. Psychol.* 24 1–24. 10.1002/ejsp.2420240102

[B19] Fredrickson B. L. (2001). The role of positive emotions in positive psychology: The Broaden-and-build theory of positive emotions. *Am. Psychol.* 56 218–226. 10.1037/0003-066X.56.3.218 11315248 PMC3122271

[B20] Fredrickson B. L. (2003). The value of positive emotions. *Am. Sci.* 91 330–335. 10.1511/2003.4.330

[B21] Furnham A. (2016). The relationship between cognitive ability, emotional intelligence and creativity. *Psychology* 7 193–197. 10.4236/psych.2016.72021

[B22] Gherardi S. Nicolini D. Odella F. (1998). Toward a social understanding of how people learn in organizations: The notion of situated curriculum. *Manage. Learn* 29 273–297. 10.1177/1350507698293002

[B23] Giselle R. (2020). Robot will take your job: Innovation for an era of artificial intelligence. *J. Bus. Res.* 116 68–74. 10.1016/j.jbusres.2020.05.019

[B24] Grzywacz J. G. Marks N. F. (2000). Reconceptualizing the work–family interface: An ecological perspective on the correlates of positive and negative spillover between work and family. *J. Occup. Health Psychol.* 5 111–126. 10.1037/1076-8998.5.1.111 10658890

[B25] Haans R. F. J. Pieters C. He Z. L. (2016). Thinking about U: Theorizing and testing U- and inverted U-shaped relationships in strategy research. *Strateg. Manage. J.* 37 1177–1195.

[B26] Hackman J. R. Oldham G. R. (1976). Motivation through the design of work: Test of a theory. *Organ. Behav. Hum. Perform.* 16 250–279. 10.1016/0030-5073(76)90016-7

[B27] Haenlein M. Kaplan A. (2019). A brief history of artificial intelligence: On the past, present, and future of artificial intelligence. *Calif. Manage. Rev.* 61 5–14. 10.1177/0008125619864925

[B28] Hancock P. A. Billings D. R. Schaefer K. E. Chen J. Y. de Visser EJ Parasuraman R. (2011). A meta-analysis of factors affecting trust in human-robot interaction. *Hum. Factors* 53 517–527. 10.1177/0018720811417254 22046724

[B29] Harman H. H. (1976). *Modern factor analysis*, 3rd revised Edn. Chicago IL: University of Chicago Press.

[B30] Hedström P. Swedberg R. (1998). *Social mechanisms: An analytical approach to social theory.* Cambridge: Cambridge University Press. 10.1017/CBO9780511663901

[B31] Hentout A. Aouache M. Maoudj A. Akli I. (2019). Human–robot interaction in industrial collaborative robotics: A literature review of the decade 2008–2017. *Adv. Robot.* 33 764–799. 10.1080/01691864.2019.1636714

[B32] Howard J. L. Gagné M. Bureau J. S. (2017). Testing a continuum structure of self-determined motivation: A meta-analysis. *Psychol. Bull.* 143 1346–1377. 10.1037/bul0000125 29048175

[B33] Hwang J. Park T. Hwang W. (2013). The effects of overall robot shape on the emotions invoked in users and the perceived personalities of robot. *Appl. Ergon.* 44 459–471. 10.1016/j.apergo.2012.10.010 23157974

[B34] Kahya E. (2007). The Effect of Job characteristics and working conditions on job performance. *Int. J. Ind. Ergon.* 37 515–523. 10.1016/j.ergon.2007.02.006

[B35] Karasek R. A. (1979). Job demands, job decision latitude, and mental strain: Implications for job redesign. *Adm. Sci. Q.* 24 285–308. 10.2307/2392498 30315367

[B36] Karasekr R. A. Theorellt T. (1990). *Healthy work: Stress, productivity, and the reconstruction of working life.* New York, NY: Basic Book.

[B37] Kemboi A. Biwott G. Chenuos N. Rutto A. (2013). Skill variety, feedback and employee performance: A case of moi teaching and referral hospital eldoret. *Eur. J. Bus. Manage.* 5 151–156.

[B38] Kulic D. Croft E. A. (2007). Affective state estimation for human-robot interaction. *IEEE Trans. Robot.* 23 991–1000. 10.1109/TRO.2007.904899

[B39] LePine J. A. Podsakoff N. P. LePine M. A. (2005). A meta-analytic test of the challenge stressor-hindrance stressor framework: An explanation for inconsistent relationships among stressors and performance. *Acad. Manage. J.* 48 764–775. 10.5465/amj.2005.18803921

[B40] Madhavan P. Wiegmann D. A. (2007). Similarities and differences between human–human and human–automation trust: An integrative review. *Theor. Issues Ergon. Sci.* 8 277–301. 10.1080/14639220500337708

[B41] Mahzoon H. Ogawa K. Yoshikawa Y. Tanaka M. Ogawa K. Miyazaki R. (2019). Effect of self-representation of interaction history by the robot on perceptions of mind and positive relationship: A case study on a home-use robot. *Adv. Robot.* 33 1112–1128. 10.1080/01691864.2019.1676823

[B42] Malhotra N. K. Kim S. S. Patil A. (2006). “Common method variance in IS research: A comparison of alternative approaches and a reanalysis of past research. *Manage. Sci.* 52 1865–1883. 10.1287/mnsc.1060.0597 19642375

[B43] Manyika J. Chui M. Miremadi M. Bughin J. George K. Willmott P. (2017). *A Future that works: Automation, employment, and productivity.* San Francisco, CA: McKinsey Global Institute.

[B44] Markoff J. A. (2016). *Machines of loving grace: The quest for common ground between humans and robots.* New York, NY: HarperCollins.

[B45] Medcof J. W. (1996). The job characteristics of computing and non-computing work activities. *J. Occup. Organ. Psychol.* 69 199–212. 10.1111/j.2044-8325.1996.tb00610.x

[B46] Michael L. Sabrina S. (2021). Decision augmentation and automation with artificial intelligence: Threat or opportunity for managers? *Bus. Horiz.* 64 711–724. 10.1016/j.bushor.2021.02.026

[B47] Miller J. B. I Stiver P. (1997). *The healing connection: How women form relationships in therapy and in life.* Boston, MA: Beacon Press.

[B48] Morris M. Venkatesh V. (2010). Job Characteristics and job satisfaction: Understanding the Role of enterprise resource planning system implementation. *MIS Q.* 34 143–161. 10.2307/20721418

[B49] Niessen C. Sonnentag S. Sach F. (2010). “Thriving at work: A diary study,” in *Working paper.* (Konstanz: University of Konstanz). 10.1037/e518392013-197

[B50] Nix G. Ryan R. M. Manly J. B. Deci E. L. (1999). Revitalization through self-regulation: The effects of autonomous and controlled motivation on happiness and vitality. *J. Exp. Soc. Psychol.* 25 266–284. 10.1006/jesp.1999.1382

[B51] Noefer K. Stegmaler R. Molter B. Sonntag K. (2009). A great many things to do and not a minute to spare:Can feedback from supervisors moderate the relationship between skill variety, time pressure and employees’ innovative behavior? *Creat. Res. J.* 21 384–393. 10.1080/10400410903297964

[B52] Oldham G. R. Cummings A. (1996). Employee creativity: Personal and contextual factors at work. *Acad. Manage. J.* 39 607–634. 10.5465/256657 256657

[B53] Papadopoulos F. Küster D. Corrigan L. J. (2016). Do relative positions and proxemics affect the engagement in a human-robot collaborative scenario?. Interaction studies. *Soc. Behav. Commun. Biol. Artif. Syst.* 17 321–347. 10.1075/is.17.3.01pap 33486653

[B54] Parasuraman S. Alutto J. A. (1984). Sources and outcomes of stress in organizational settings: Toward the development of a structural model. *Acad. Manage. J.* 27 330–350. 10.5465/255928

[B55] Paulsson K. Ivergård T. Hunt B. (2005). Learning at work: Competence development or competence-stress. *Appl. Ergon.* 36 135–144. 10.1016/j.apergo.2004.09.008 15694067

[B56] Podsakoff P. M. MacKenzie S. B. Lee J.Y. (2003). Common method bias in behavioral research: A critical review of the literature and recommended remedies. *J. Appl. Psychol*. 88:903. 10.1037/0021-9010.88.5.879 14516251

[B57] Porath C. Spreitzer G. Gibson C. Garnett F. G. (2012). Thriving at work: Toward its measurement, construct validation, and theoretical refinement. *J. Organ. Behav.* 33 250–275. 10.1002/job.756

[B58] Preacher K. J. Hayes A. F. (2008). Asymptotic and resampling strategies for assessing and comparing indirect effects in multiple mediator models. *Behav. Res. Methods* 40 879–891. 10.3758/BRM.40.3.879 18697684

[B59] Prem R. Kubicek B. Diestel S. Korunka C. (2016). Regulatory job stressors and their within-person relationships with ego depletion: The roles of state anxiety, self-control effort, and job autonomy. *J. Vocat. Behav.* 92 22–32. 10.1016/j.jvb.2015.11.004

[B60] Prem R. Ohly S. Kubicek B. Korunka C. (2017). Thriving on challenge stressors? Exploring time pressure and learning demands as antecedents of thriving at work. *J. Organ. Behav.* 38 108–123. 10.1002/job.2115 28133415 PMC5244684

[B61] Rahwan I. Cebrian M. Obradovich N. Bongard J. Bonnefon J.-F. Breazeal C. (2019). Machine behavior. *Nature* 568 477–486. 10.1038/s41586-019-1138-y 31019318

[B62] Rampersad G. (2020). Robot will take your job: Innovation for an era of artifificial intelligence. *J. Bus. Res.* 116 68–74.

[B63] Riek B. M. Mania E. W. Gaertner S. L. (2006). Intergroup threat and outgroup attitudes: A meta-analytic review. *Pers. Soc. Psychol. Rev.* 10 336–353. 10.1207/s15327957pspr1004_4 17201592

[B64] Ryan R. M. Deci E. L. (2000). Self-determination theory and the facilitation of intrinsic motivation, social development, and well-being. *Am. Psychol*. 55 68–78. 10.1037/0003-066X.55.1.68 11392867

[B65] Salanova M. Schaufeli W. B. (2008). A cross-national study of work engagement as a mediator between job resources and proactive behaviour. *Int. J. Hum. Resour. Manage*. 19 116–131. 10.1080/09585190701763982

[B66] Seo M. G. Barrett L. F. Bartunek J. M. (2004). The role of affective experience in work motivation. *Acad. Manage. Rev.* 29 423–439. 10.2307/20159052 16871321 PMC1519413

[B67] Sheldon K. M. Elliot A. J. Kim Y. Kasser T. (2001). What is satisfying about satisfying events? Testing candidate psychological needs. *J. Pers. Soc. Psychol.* 80 325–339. 10.1037/0022-3514.80.2.325 11220449

[B68] Sims H. P. Szilagyi A. D. Keller R. T. (1976). The measurement of job characteristics. *Acad. Manage. J.* 19 195–212. 10.2307/255772 1029346

[B69] Sonnentag S. Fritz C. (2007). The recovery experience questionnaire: Development and validation of a measure assessing recuperation and unwinding at work. *J. Occup. Health Psychol.* 12 204–221. 10.1037/1076-8998.12.3.204 17638488

[B70] Spreitzer G. M. Lam C. F. Fritz C. (2010). “Engagement and human thriving: Complementary perspectives on energy and connections to work,” in *Work engagement: A handbook of essential theory and research*, eds Bakker A. B. Leiter M. (New York, NY: Psychology Press), 132–146.

[B71] Spreitzer G. Porath C. (2013). “Self-determination as nutriment for thriving: Building an integrative model of human growth at work,” in *Oxford Handbook of Work Engagement, Motivation, and Self-Determination Theory*, ed. Gagné M. (New York, NY: Oxford University Press).

[B72] Spreitzer G. Sutcliffe K. Dutton J. Sonenshein S. Grant A. M. (2005). A socially embedded model of thriving at work. *Organ. Sci.* 16 537–549. 10.1287/orsc.1050.0153 19642375

[B73] Taks M. Tynjala P. Toding M. Kukemelk H. Venesaar U. (2014). “Engineering students’ experiences in studying entrepreneurship. *J. Eng. Educ.* 103 573–598. 10.1002/jee.20056

[B74] Thomas L. T. Ganster D. C. (1995). Impact of family supportive work variables on work–family conflict and strain: A control perspective. *J. Appl. Psychol.* 80 6–15. 10.1037/0021-9010.80.1.6

[B75] Thompson C. A. Prottas D. J. (2006). Relationships among organizational family support, job autonomy, perceived control, and employee well-being. *J. Occup. Health Psychol.* 11:100. 10.1037/1076-8998.10.4.100 16551178

[B76] Uphill M. A. Rossato C. Swain J. O’Driscoll J. (2019). Challenge and threat: A critical review of the literature and an alternative conceptualization. *Front. Psychol.* 10:1255. 10.3389/fpsyg.2019.01255 31312151 PMC6614335

[B77] van Esch P. Stewart Black J. Franklin D. Harder M. (2021). AI-enabled biometrics in recruiting: Insights from marketers for managers. *Australas. Mark. J.* 29 225–234. 10.1016/j.ausmj.2020.04.003

[B78] Vergauwe J. Wille B. Feys M. De Fruyt F. Anseel F. (2015). Fear of being exposed: The trait-relatedness of the impostor phenomenon and its relevance in the work context. *J. Bus. Psychol.* 30 565–581. 10.1007/s10869-014-9382-5

[B79] Visser E. Parasuraman R. (2011). Adaptive aiding of human-robot teaming. *J. Cogn. Eng. Decis. Mak.* 5 209–231. 10.1177/1555343411410160

[B80] Voydanoff P. (2004). The effects of work demands and resources on work-to-family conflict and facilitation. *J. Marriage Fam.* 66 398–412. 10.1111/j.1741-3737.2004.00028.x

[B81] Waschull S. Bokhorst J. A. C. Molleman E. Wortmann J. C. (2020). Work design in future industrial production: Transforming towards cyberphysical systems. *Comput. Ind. Eng.* 139:105679. 10.1016/j.cie.2019.01.053

[B82] Wenger E. (1998). *Communities of practice: Learning, meaning and identity.* New York, NY: Cambridge University Press. 10.1017/CBO9780511803932

[B83] Yang Y. Rui Y. Yan M. (2022). Can’ t disconnect even after-hours: How work connectivity behavior after-hours affects employees’ thriving at work and family. *Front. Psychol.* 13:865776. 10.3389/fpsyg.2022.865776 35356326 PMC8959651

[B84] Zlotowski J. Yogeeswaran K. Bartneck C. (2017). Can we control it? Autonomous robots threaten human identity, uniqueness, safety, and resources. *Int. J. Hum. Comput. Stud.* 100 48–54. 10.1016/j.ijhcs.2016.12.008

